# Stabilization of the V2 loop improves the presentation of V2 loop–associated broadly neutralizing antibody epitopes on HIV-1 envelope trimers

**DOI:** 10.1074/jbc.RA118.005396

**Published:** 2019-02-06

**Authors:** Steven W. de Taeye, Eden P. Go, Kwinten Sliepen, Alba Torrents de la Peña, Kimberly Badal, Max Medina-Ramírez, Wen-Hsin Lee, Heather Desaire, Ian A. Wilson, John P. Moore, Andrew B. Ward, Rogier W. Sanders

**Affiliations:** From the ‡ Department of Medical Microbiology, Amsterdam UMC, University of Amsterdam, Amsterdam, 1105 AZ, The Netherlands,; the § Department of Chemistry, University of Kansas, Lawrence, Kansas 66045,; the ¶ Department of Integrative Structural and Computational Biology, Scripps CHAVI-ID, IAVI Neutralizing Antibody Center and Collaboration for AIDS Vaccine Discovery, The Scripps Research Institute, La Jolla, California 92037, and; the ‖ Department of Microbiology and Immunology, Weill Medical College of Cornell University, New York, New York 10021

**Keywords:** human immunodeficiency virus (HIV), glycoprotein structure, structure-function, protein design, antibody, vaccine development, HIV-1 envelope glycoprotein trimer

## Abstract

A successful HIV-1 vaccine will likely need to elicit broadly neutralizing antibodies (bNAbs) that target the envelope glycoprotein (Env) spike on the virus. Native-like recombinant Env trimers of the SOSIP design now serve as a platform for achieving this challenging goal. However, SOSIP trimers usually do not bind efficiently to the inferred germline precursors of bNAbs (gl-bNAbs). We hypothesized that the inherent flexibilities of the V1 and V2 variable loops in the Env trimer contribute to the poor recognition of gl-bNAb epitopes at the trimer apex that extensively involve V2 residues. To reduce local V2 flexibility and improve the binding of V2-dependent bNAbs and gl-bNAbs, we designed BG505 SOSIP.664 trimer variants containing newly created disulfide bonds intended to stabilize the V2 loop in an optimally antigenic configuration. The first variant, I184C/E190C, contained a new disulfide bond within the V2 loop, whereas the second variant, E153C/R178C, had a new disulfide bond that cross-linked V2 and V1. The resulting engineered native-like trimer variants were both more reactive with and were neutralized by V2 bNAbs and gl-bNAbs, a finding that may be valuable in the design of germline targeting and boosting trimer immunogens to create an antigenic conformation optimal for HIV vaccine development.

## Introduction

There remains an urgent need to create an effective vaccine to reduce the further spread of HIV-1. Many vaccine design strategies are now based on the elicitation of broadly neutralizing antibodies (bNAbs)^2^ that recognize the envelope glycoprotein spike (Env) on the surface of infectious viruses (1, 2). Achieving this goal is a formidable challenge that requires harnessing structure-based Env immunogen design and an increased understanding of the immunology and genetics of how bNAbs evolve in a subset of infected individuals (3, 4). A now widely used immunogen development platform is based on native-like, recombinant soluble trimers of the SOSIP design (5, 6).

The native Env trimer is embedded in the HIV-1 membrane and is composed of three gp41 subunits that are noncovalently linked to three gp120 subunits (7, 8). Each gp120 subunit is composed of five conserved domains (C1–C5) and five variable domains (V1–V5) that are shielded by 25–35 *N*-linked glycans. Three or four additional glycans are present on ectodomain of gp41 (9). Nine highly conserved disulfide bonds help to maintain gp120 in an appropriate configuration, and another disulfide bond is present within the gp41 ectodomain (10, 11). The gp120 disulfide bonds are mostly located in the core structure from which the variable loops and *N*-linked glycans protrude (8, 12, 13). The stems of the V1, V2, V3, and V4 loops are all stabilized by disulfide bonds. Some HIV-1 isolates contain additional cysteine residues within the V2 and/or V4 loops, allowing the formation of a tenth and, in rare cases, an eleventh disulfide within the gp120 subunit (14–18). An additional intra-V2 disulfide is frequently present in HIV-2 and SIV gp120 proteins (18). When gp120 monomers or nonnative gp140 proteins are expressed as recombinant proteins, “scrambling” of some disulfide bonds, particularly in the V1V2 region, generally occurs, leading to the production of misfolded proteins (19, 20). This problem does not, however, apply to SOSIP trimers (21, 22).

Multiple bNAbs target an epitope cluster centered on the V2 domain at the trimer apex, including PG9, PG16, PGT145, PGDM1400, CH01, PCT64, VRC38, and VRC26.09 (from hereon termed V2 bNAbs) (23–25). In general, the V2 bNAbs recognize one or two of the glycans at positions 156 and 160, together with a relatively conserved peptide sequence: the positively charged β-strand C (residues 166–176) (26–35). Components from two or three gp120 monomers are required to fully form these epitopes. Hence the V2 bNAbs are either truly quaternary in nature (trimer-specific) or trimer-preferring. Their binding to β-strand C is frequently mediated via an unusually long CDRH3 loop that penetrates a small gap between the glycans at positions 156 and 160 (26–35). The V2 bNAbs are of interest for Env immunogen design because they are elicited relatively frequently and rather sooner during HIV-1 infection compared with other bNAb classes (36–38). Accordingly, their maturation has been found to require a comparatively low percentage of somatic hypermutation (27, 31, 39–41).

As noted above, the BG505 and other genotypes of the SOSIP.664 native-like trimer now serve as platforms for structure-guided immunogen design as part of bNAb-development programs (8, 12, 13, 42–45). One fruitful avenue of exploration is to improve how SOSIP trimers present specific bNAb epitopes to naïve B cells by stabilizing them. The V2 bNAb epitope cluster is particularly attractive from this perspective in view of the exceptional flexibility of this region of the trimer (26, 46, 47), which might hinder a productive interaction with the appropriate B-cell receptors (BCRs) on naïve B cells and thus not lead to effective B cell activation and affinity maturation. Rigidifying the V2, and also the nearby V1, might also reduce the extent to which these loops shield other bNAb epitopes, such as those associated with the CD4bs (48–52). Thus, several studies have linked the length of the V1 and V2 loops with the neutralization sensitivity of viruses, longer loops with a concomitantly increased number of glycan sites being associated with resistance (14, 49, 52–54).

The availability of high-resolution structural information on the architecture of BG505 and other SOSIP trimers is a critical guide to design improvements, including those to the V2 bNAb epitopes. The V1V2 domain folds into a four stranded anti-parallel β-sheet Greek key motif in which strands A and B are connected by the flexible V1 loop and strands C and D by the flexible V2 loop (8, 12, 13, 26, 43). Interactions between the V1V2 domains of different gp120 subunits help to stabilize the trimer apex and thereby the entire trimer; additional interactions with the V3 domain stabilize the prefusion conformation of the trimer, in which V3 domain is sequestered underneath the V1V2 loops (13, 43, 44, 55, 56). The overall flexibility of the V1V2 domains on the prefusion trimer is indicated by the lack of density for many residues and the high B-values for others in this area of the trimer (8, 12, 13, 43–45, 57, 58). Hydrogen deuterium exchange analyses of SOSIP trimers provide additional information on comparatively unusual flexibility of the V1 and V2 loop regions (46, 47).

Here, we sought to stabilize the V1 and V2 domains by introducing disulfide bonds either within the V2 loop or between the V1 and V2 loops. We show that both strategies can improve the presentation of V2 bNAb epitopes. Thus, the resulting SOSIP trimer variants have improved reactivity with V2 bNAbs and their inferred germline (gl) precursors. Moreover, the corresponding virus mutants are more sensitive to neutralization by V2 bNAbs. These trimer-improvement methods may therefore play a role in bNAb-induction strategies.

## Results

### Design of V2-internal and V1-V2 disulfide bonds at the trimer apex

**Figure 1.**
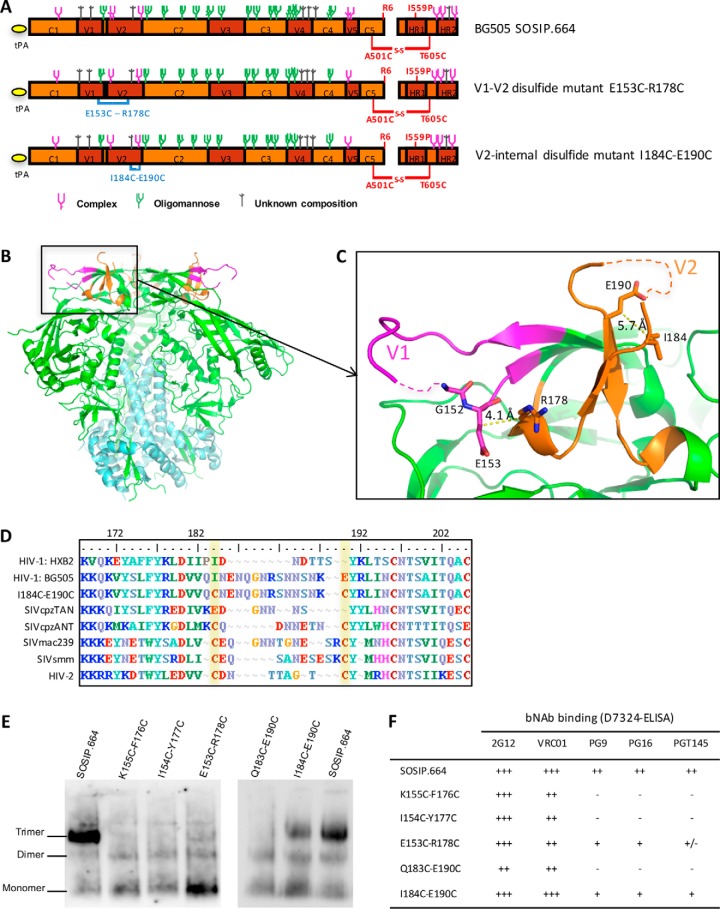
**Design and preliminary characterization of BG505 SOSIP.664 disulfide bond mutants.** *A*, linear representation of BG505 SOSIP.664 and its V1-V2 and V2-internal disulfide bond mutants. The engineered disulfide bonds are indicated in *blue*. The glycan composition of the SOSIP.664 trimer is derived from Ref. 9. *B*, crystal structure of the BG505 SOSIP.664 trimer (PDB code 5CEZ (44)). The protomers are colored as follows: gp41 subunit in *cyan*, V1 loop in *purple*, V2 loop in *orange*, and other gp120 domains in *green. C*, detailed view of the V1V2 region. Residues that are changed to a cysteine in the V1-V2 disulfide bond mutants are shown as *sticks*. The distances (in Å) between the β carbons (Cβ) of the mutated residue pairs are given next to the *yellow dotted lines. D*, alignment of the V2 region (residues 168–205) of several HIV-1, SIV and HIV-2 sequences (adapted from Ref. 18). Residues 184 and 190 (HxB2 numbering) are highlighted in *yellow* to show the cysteine pair (*i.e.* presumed V2-internal disulfide bond) that is present in SIV and HIV-2 sequences but absent from HIV-1. *E*, the indicated 293T cell-expressed SOSIP.664 and disulfide mutant proteins were analyzed by BN-PAGE followed by Western blotting with bNAb 2G12. *F*, summary of bNAb binding of the same SOSIP.664 proteins. The binding efficiency, based on the area under the curve (AUC) values, is indicated by the following code: +++, very strong binding; ++, strong binding; +, moderate binding; +/−, weak binding; −, no binding.

We designed disulfide bond mutants to stabilize the flexible V1 and V2 domains on BG505 SOSIP.664 trimers (Fig. 1, *A–C*). To stabilize both domains simultaneously, we constructed three mutants, K155C/F176C, I154C/Y177C, and E153C/R178C, in which the C-terminal part of V1 is now linked to the N-terminal part of V2 by an engineered interloop disulfide bond (Fig. 1*C*). This category of mutant is hereafter referred to by the term V1-V2 disulfide bond. The positions for the Cys substitutions were chosen because of the close proximity of residues Lys^155^ and Phe^176^ (5.5 Å distance between the β-carbons (Cβ) atoms; PDB code 5CEZ), Ile^154^ and Tyr^177^ (5.4 Å), and Glu^153^ and Arg^178^ (4.1 Å); at such distances, it seemed feasible that a disulfide bond could be formed (44) (Fig. 1*C*).

We also elected to stabilize the V2 loop by creating a new disulfide bond within it. Accordingly, we designed two mutants: Q183C/E190C and I184C/E190C (Fig. 1, *A–C*). This category of mutant is hereafter referred to by the term V2-internal disulfide bond. The positions for the Cys substitutions were chosen because a twin cysteine motif is present at the corresponding positions in the V2 loops of SIV and HIV-2 Env and has been suggested to lead to the formation of a loop-stabilizing disulfide bond (Fig. 1*D*) (18). As for the V1-V2 disulfide bonds, we used the SOSIP trimer structure to show that residues 184 and 190 are in close proximity (Cβ distance of 5.7 Å), which favors disulfide bond formation, whereas residues 183 and 190 are slightly farther apart (6.4 Å; Fig. 1*C*).

The five disulfide bond mutants described above were introduced into a D7324-tagged BG505 SOSIP.664 construct (Fig. 1, *A–C*), and the resulting proteins were expressed transiently in 293T cells. We then assessed the yield, trimer formation, and antigenicity of each mutant, using the unpurified culture supernatants. All three V1-V2 disulfide mutants were expressed at acceptable yields (Fig. S1*A*), but a BN-PAGE gel showed that trimer formation was severely impaired (Fig. 1*E*). The implication is that the disulfide bond–linked V1V2 domains are no longer able to form the intersubunit interactions at trimer apex that are important for trimer formation. The conformationally nonselective 2G12 and VRC01 bNAbs bound the various V1-V2 disulfide bond mutant proteins efficiently (Fig. 1*F* and Fig. S1*B*). However, the conformationally sensitive (trimer-specific or trimer-preferring) bNAbs PG9, PG16, and PGT145 failed to bind to the K155C/F176C and I154C/Y177C mutants and reacted only poorly with the E153C/R178C mutant. These data patterns are consistent with the poor formation of trimers seen in the gel-based analysis (Fig. 1*F* and Fig. S1*B*).

The V2-internal disulfide bond mutants were both slightly less efficiently expressed than the parental BG505 SOSIP.664 construct (Fig. S1*A*). The Q183C/E190C mutant failed to form trimers, but a moderate level of trimer formation was observed for the I184C/E190C mutant (Fig. 1*E*). In the antigenicity studies, the Q183C/E190C mutant bound 2G12 and VRC01 somewhat less well than the SOSIP.664 comparator and did not bind PG9, PG16, and PGT145 at all (Fig. 1*F* and Fig. S1*C*). In contrast, the I184C/E190C mutant bound comparably to 2G12 and VRC01 and was also reactive with PG9, PG16, and PGT145, albeit to a lesser extent than SOSIP.664 (Fig. 1*F* and Fig. S1*C*). We judged that the extent of binding of these three V2 bNAbs was sufficient to pursue further studies of this V2-internal disulfide bond mutant (see below).

### Evolutionary rescue of the V1-V2 disulfide bond mutants in LAI virus

Although the K155C/F176C, I154C/Y177C, and E153C/R178C V1-V2 disulfide bond mutants formed trimers very inefficiently, we decided to explore whether their defects could be improved by using a virus evolution procedure. To do so, we introduced each of the three cysteine pairs into a HIV-1 LAI molecular clone, because this virus has been used successfully to repair Env folding defects caused by changes in the V1V2 domain and/or the overall disulfide bond architecture (59–62). The LAI clone we used was engineered to introduce four amino acids that create the PG9 epitope, as judged by neutralization sensitivity (see “Materials and methods” for details).

**Figure 2.**
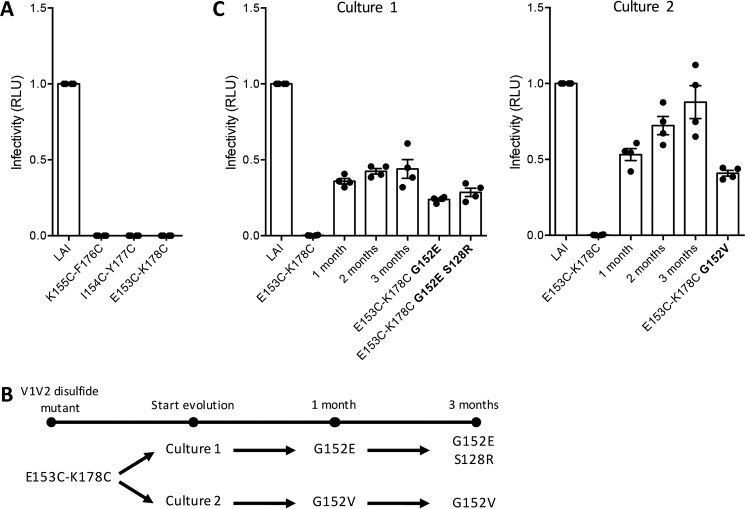
**Evolutionary repair of V1-V2 disulfide bond mutant LAI viruses.** *A*, relative infection of TZM-bl cells by the indicated parental and disulfide bond mutant LAI viruses, as measured by a luciferase assay. *B*, schematic timeline showing the mutations that were found over time in SupT1 evolution cultures 1 and 2 that were infected by the E153C/K178C V1-V2 disulfide mutant. *C*, infection of TZM-bl cells by the LAI-PG9 virus; the E153C/K178C mutant; supernatants from the SupT1 evolution cultures taken after 1, 2, or 3 months; and the E153C/K178C virus variants containing the compensatory changes identified in the evolution cultures (changes indicated in *bold*). *Left panel*, evolution culture 1; *right panel*, evolution culture 2. The mean infectivity of four individual measurements is depicted in all graphs, in which individual measurements are shown as *dots*.

As expected from the studies on the BG505 trimers, none of the three V1-V2 disulfide bond mutant LAI viruses was infectious, as measured in a single round infection assay in TZM-bl cells (Fig. 2*A*). Each mutant virus was therefore passaged for a prolonged period in two independent SupT1 T cell cultures to see whether compensatory mutations arose that increased replication efficiency. The K155C/F176C and I154C/Y177C mutants failed to replicate detectably during the 3-month experiment, indicating that their defects were beyond repair. However, the E153C/K178C mutant acquired replication competence in both cultures after 3 weeks (note that position 178 in the LAI clone is a lysine and not the arginine present at the corresponding position in BG505 Env). Proviral DNA was therefore sequenced after 1, 2, and 3 months of culture. In both cultures, a point substitution at position 152 was found to have emerged within 1 month (Fig. 2*B*). Specifically, glycine 152 had changed to either glutamic acid (G152E; culture 1) or valine (G152V; culture 2). The significance of these observations is that residue 152 is directly adjacent to the cysteine substitution at position 153. The changes at position 152 persisted to the end of the 3-month culture period, but we also found that a second change, S128R, arose in the virus in culture 1 (Fig. 2*B*).

We next assessed the ability of the E153C/K178C virus quasispecies from the SupT1 cultures to infect TZM-bl cells and found that there was a gradual gain of infectivity during the 3 months of evolutionary rescue. By the end of the culture period, the E153C/K178C mutant virus in SupT1 cultures 1 and 2 showed infectivity levels of ∼50 and ∼100% compared with the parental LAI-PG9 virus, respectively (Fig. 2*C*). To confirm that the G152E, G152V, and S128R compensatory mutations identified by sequencing the *env* genes were in fact responsible for the restored infectivity, we introduced these changes individually or in combination into an LAI clone that also contained the Cys^153^–Cys^178^ V1-V2 disulfide bond. In all three cases, the resulting virus mutants and the quasispecies present in the evolution cultures at month 3 had comparable infectivities for TZM-bl cells (Fig. 2*C*). The substitutions at position 152 had the strongest effect, whereas the S128R change modestly increased the infectivity of the G152E mutant. Taken together, the evolutionary repair experiments show that a compensatory substitution at position 152, either G152E or G152V, partially restores the infectivity of an otherwise replication-defective E153C/K178C V1-V2 disulfide bond mutant LAI virus.

### The G152E substitution improves trimer formation by the BG505 SOSIP.664 E153C/R178C V1-V2 disulfide bond mutant

**Figure 3.**
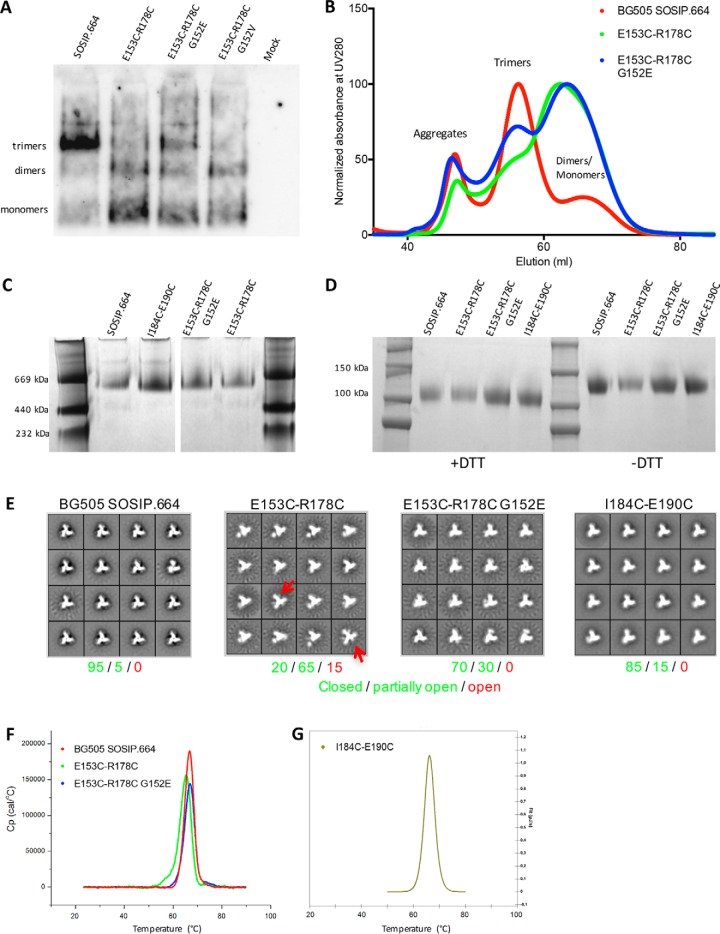
**Biochemical analysis of BG505 SOSIP.664 disulfide mutants.** *A*, BN-PAGE and Western blotting analysis of culture supernatants from 293T cells expressing the SOSIP.664 or V1-V2 disulfide mutant proteins. *B*, SEC chromatogram of the indicated SOSIP.664 and V1-V2 disulfide mutant proteins, isolated by 2G12 affinity chromatography from 293T cell culture supernatants. *C*, Coomassie Blue–stained BN-PAGE gel of the PGT145-purified SOSIP.664 and disulfide mutant proteins. *D*, Coomassie Blue–stained reducing (+ *DTT*) or nonreducing (− *DTT*) SDS-PAGE gels of the same PGT145-purified trimers. *E*, 2D class averages of NS-EM analysis of the indicated PGT145-purified disulfide mutant trimers. NS-EM analysis of 2G12–SEC-purified BG505 SOSIP.664 was used as control, which shows a similar closed native-like trimer conformation as previously described (47, 65). The percentages of closed native-like, partially open native-like, and fully open trimers (64) are shown in *green*, *green*, and *red*, respectively (Table 1). The *red arrows* indicate fully open E153C/R178C V1-V2 disulfide mutant trimers that resemble the CD4-bound conformation of the SOSIP.664 trimer (CD4 was not present in this experiment). *F*, thermal melting profiles for the indicated PGT145-purified SOSIP.664 or V1-V2 disulfide mutant proteins obtained by DSC. *G*, DSC profile for the I184C/E190C V2-internal disulfide mutant.

To assess whether the LAI compensatory mutations at residue 152 would translate to the BG505 SOSIP.664 trimer context, we introduced either the G152E or G152V change into the SOSIP.664 E153C/R178C mutant and expressed the proteins by transiently transfecting 293T cells. The G152E compensatory mutation clearly improved both trimer formation, judged using a BN-PAGE gel and the binding of the conformationally sensitive V2 bNAbs (Fig. 3*A* and Fig. S2*B*). However, the G152V mutation was not beneficial (Fig. 3*A* and Fig. S2*B*).

We then expressed the BG505 SOSIP.664 WT, E153C/R178C, and E153C/R178C/G152E constructs in 293F cells, purified the resulting Env proteins via 2G12 affinity chromatography, and analyzed them with size-exclusion chromatography (SEC). 2G12 binds to all possible forms of Env (aggregates, trimers, dimers, and monomers), and SEC allowed us to determine the trimer formation efficiency for each Env protein. Compared with SOSIP.664, the SEC profile of the E153C/R178C mutant contained many more monomers and dimers and proportionately far fewer trimers, but trimer formation was clearly increased when the G152E change was also present (Fig. 3*B*), confirming the results of the HEK 293T transfection (Fig. 3*A*).

### Purification and characterization of loop-stabilized BG505 SOSIP.664 trimers

The improvement described above was not sufficient to allow the BG505 SOSIP.664 E153C/R178C/G152E trimers to be purified by the 2G12/SEC method. However, PGT145-affinity chromatography is an alternative approach to isolate native-like trimers (47, 63). We therefore used this method to purify trimers from the SOSIP.664 E153C/R178C (± G152E) constructs and also the I184C/E190C V2-internal disulfide mutant. In each case, trimers were successfully isolated, but at markedly variable yields (Fig. 3*C*). Whereas the trimer yield for the BG505 SOSIP.664 construct was ∼2.0 mg/liter, the corresponding values for the E153C/R178C and E153C/R178C/G152E variants were ∼0.2 and ∼0.6 mg/liter, respectively (Table 1). The yield of I184C/E190C V2-internal disulfide bond mutant trimers was ∼0.8 mg/liter (Table 1). As expected, all of the PGT145-purified trimer variants were cleaved, judged using reducing and nonreducing SDS-PAGE gels (Fig. 3*D*).

**Table 1:** **Biochemical characterization of BG505 SOSIP.664 disulfide mutant trimers** The biophysical properties of 293F cell-expressed, PGT145-purified SOSIP.664-D7324 trimers were assessed using NS-EM to determine native-like trimer formation and by DSC to quantify thermostability (*T*_m_). The unprocessed EM data are shown in Fig. 3*E*. The DSC data were fitted using a two-state model (Fig. 3, *F* and *G*).

Construct (BG505 SOSIP.664)	Yield	NS-EM				DSC	
		Native-like trimers	Closed	Partially open	Open	*T*_m_	Δ*T*_m_
	*mg/liter*	%				°*C*	°*C*
Wildtype	2.0	>95	95	5	0	66.6	
E153C/R178C	0.2	>95	20	65	15	65.3	−1.3
E153C/R178C/G152E	0.6	>95	70	30	0	66.7	+0.1
I184C/E190C	0.8	>95	85	15	0	66.3	−0.3

To confirm that the engineered disulfide bonds had actually formed, the loop-stabilized BG505 SOSIP.664 trimers were trypsin-digested into fragments that were analyzed by MS to identify peptides linked by disulfide bonds (21). The E153C/R178C V1-V2 and I184C/E190C V2-internal disulfide bonds were successfully identified among the respective trimer-derived peptides (Fig. S3*A*). Alternative (*i.e.* aberrantly formed) disulfide bonds were also detected in low abundance, but they were quantitatively and qualitatively similar to ones previously identified in the WT BG505 SOSIP.664 trimer (21). We conclude that in the PGT145-purified disulfide mutant trimers, the engineered cysteine residues do form the intended V1-V2 or V2-internal disulfide bonds, without appreciable aberrant disulfide bonds (Fig. S3*B*).

Negative-stain (NS) EM showed that the PGT145-purified SOSIP.664 E153C/R178C, E153C/R178C/G152E, and I184C/E190C disulfide bond mutant trimers were all in a native-like conformation. The SOSIP.664 trimer was in a closed, fully native-like conformation (>95%), as reported previously (Fig. 3*E*) (42). In contrast, the E153C/R178C V1-V2 disulfide bond mutant trimers, although still native-like, contained a major subpopulation (64%) that was partially open and a minor subpopulation (13%) that was fully open in an overall conformation that resembled the CD4-bound state (Table 1 and Fig. 3*E*) (64). The presence of additional density outside the trimer core resembles loop flexibility and is described to represent a partially open trimer conformation (22, 63). We have not previously seen the fully open conformation when various SOSIP trimers are PGT145-purified (47, 63). The implication is that the V1-V2 disulfide mutant is unstable and/or has a high tendency to sample more open conformations. However, the E153C/R178C/G152E mutant was mostly (68%) in the closed native-like conformation, reflecting the beneficial effect of the G152E change (Table 1 and Fig. 3*E*). The I184C/E190C V2-internal disulfide bond mutant had a very similar appearance to the SOSIP.664 parental trimer, being predominantly (86%) in the closed native-like conformation (Table 1 and Fig. 3*E*).

We used differential scanning calorimetry (DSC) to quantify the thermostability of the modified SOSIP.664 trimers. The E153C/R178C V1-V2 disulfide bond mutant was slightly less thermostable than SOSIP.664 (*T*_m_ = 65.3 °C *versus* 66.6 °C), whereas the additional presence of the G152E compensatory mutation reverted the *T*_m_ to 66.7 °C (Table 1 and Fig. 3*F*). The corresponding *T*_m_ value of 66.3 °C for the I184C/E190C V2-internal disulfide bond mutant trimer was also almost identical to SOSIP.664 (Table 1 and Fig. 3*G*). Thus, in contrast to when disulfide bonds are introduced into the core regions of a gp120 monomer (I201C/A433C) (58) or SOSIP trimer (E49C/L555C and A73C/A561C) (44, 65), engineering disulfide bonds into the V1V2 region of the SOSIP.664 trimer does not increase thermostability. The implication is that thermostability as measured by DSC is predominantly determined by the Env protein core and not by the external loops. In summary, via structure-guided design, virus evolution, and PGT145 positive selection methodologies, we were able to purify native-like BG505 SOSIP.664 trimers with an additional disulfide bond either within the V2 loop or linking the V1 and V2 loops.

### Improved binding of V1-V2 and V2-internal disulfide bond mutant trimers to V2 bNAbs and gl-bNAbs

The goal of our studies was to investigate whether we could improve the antigenicity of SOSIP.664 trimers for V2 bNAbs. We therefore tested an extensive panel of bNAbs and non-NAbs for their abilities to bind the disulfide mutant trimers in an ELISA. The various trimers were purified by PGT145 affinity chromatography as described above.

**Figure 4.**
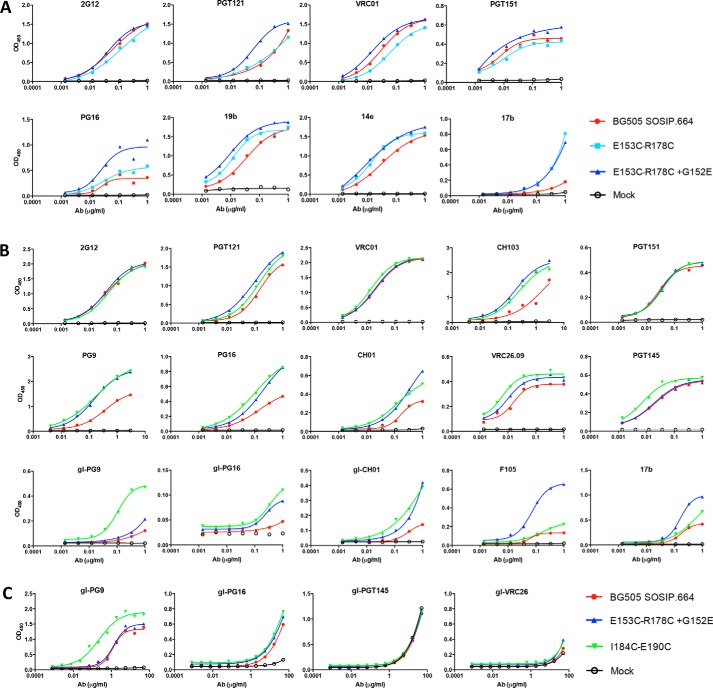
**Antigenicity of BG505 SOSIP.664 disulfide mutants.** The panels show the binding of the indicated bNAbs and non-NAbs to various PGT145-purified trimers in a D7324-capture ELISA. *A* and *B*, bNAb and non-NAb binding to SOSIP.664 (*red*), the E153C/R178C V1-V2 mutant (*light blue*), the E153C/R178C/G152E V1-V2 mutant (*dark blue*), and the I184C/E190C V2-internal disulfide mutant (*green*). *C*, the same proteins as in *B*, but with the indicated V2 gl-bNAbs tested in a modified version of the capture ELISA that was optimized for measuring gl-bNAb binding (see “Materials and methods”). The binding curves in this figure are representative of three individual experiments. The mean AUC value was used to calculate fold changes when comparing Ab binding to disulfide mutant trimers *versus* SOSIP.664. These values are listed in Table 2.

Compared with BG505 SOSIP.664, the E153C/R178C V1-V2 disulfide mutant bound the VRC01, PGT151, and 2G12 slightly less well, whereas the 19b, 14e, and 17b non-NAbs targeting V3 and CD4i epitopes bound more strongly (Fig. 4*A*). Taken together, these results imply that the more open appearance of these trimers observed in NS-EM studies is associated with an antigenically less preferred conformation. The introduction of the G152E compensatory mutation improved VRC01, PGT151, and 2G12 binding and was also extremely beneficial to the epitopes for the V3-glycan bNAb PGT121 and the V2 bNAbs PG9, PG16, and CH01; indeed, the latter four bNAbs bound better to the E153C/R178C/G152E mutant than to SOSIP.664 (Table 2 and Fig. 4, *A* and *B*). An increased binding to CD4bs bNAb CH103 was also observed for the E153C/R178C/G152E mutant (Table 2 and Fig. 4*B*). However, several non-NAbs also bound more strongly to this mutant trimer, compared with SOSIP.664 (Table 2 and Fig. 4, *A* and *B*).

**Table 2:**
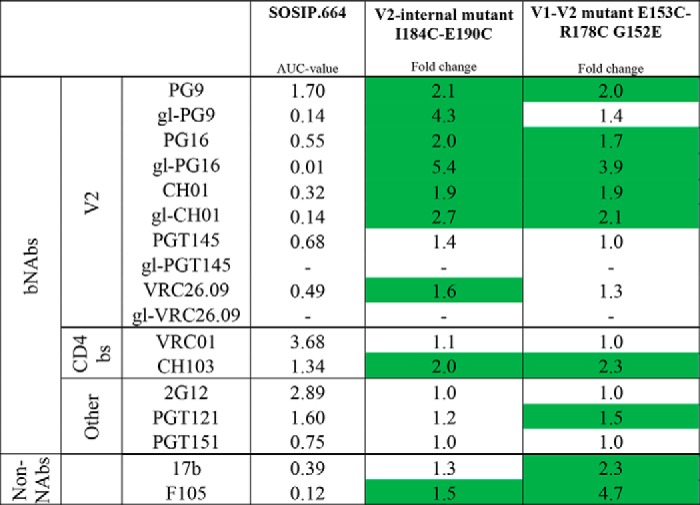
**Antigenicity of BG505 SOSIP.664 disulfide mutant trimers** The binding of bNAbs, gl-bNAbs, and non-NAbs to PGT145-purified SOSIP.664 and mutant trimers was assessed by D7324-capture ELISA (Fig. 4). The AUC values are measures of binding efficiency. The fold changes in AUC value compared to SOSIP.664 for both mutants are listed, with those ≥1.5 highlighted in green.

The I184C/E190C V2-internal disulfide bond mutant trimer bound all the V2 bNAbs (PG9, PG16, PGT145, VRC26.09, and CH01) better than SOSIP.664. The titration curves showed that the maximum binding levels were substantially higher for the mutant trimers compared with SOSIP.664 (Table 2 and Fig. 4*B*). Although binding to VRC01 was similar between mutant and SOSIP.664 trimer, binding to CH103 was improved for the V2-internal disulfide bond mutant trimer (Table 2 and Fig. 4*B*). There were no, or only very minor, differences in how the 2G12, PGT121, and PGT151 bNAbs or the F105 and 17b non-NAbs bound to the mutant and parental SOSIP.664 trimers (Table 2 and Fig. 4*B*).

We also assessed whether the gl-PG9, gl-PG16, and gl-CH01 V2 bNAbs were more reactive with the mutant trimers. As reported previously, all three gl-bNAbs bound detectably to the BG505 SOSIP.664 trimer, albeit with lower affinities than the corresponding mature bNAbs (Fig. 4*B*) (66). However, the three gl-bNAbs each bound substantially more efficiently with the I184C/E190C V2-internal disulfide mutant trimer than with SOSIP.664 (Table 2 and Fig. 4*B*). Based on half-maximal binding (EC_50_) values, gl-PG9 binding to the mutant was increased by 5-fold, and the maximum binding level was also higher (Fig. 4*C*). A similar pattern was seen for the gl-PG16 and gl-CH01 bNAbs, although EC_50_ values could not be reliably determined because the titration curves did not reach a plateau (Table 2 and Fig. 4, *B* and *C*).

The V1-V2 disulfide bond mutant trimer was slightly more reactive with gl-PG9, gl-PG16, and gl-CH01 compared with SOSIP.664, although the beneficial effects of this engineered disulfide were markedly less than those conferred by the V2-internal disulfide (Table 2 and Fig. 4*B*). None of the trimer variants bound gl-PGT145 or gl-VRC26.09 detectably (Table 2 and Fig. 4*C*).

Binding of V2 bNAbs to the V1-V2 disulfide mutant and SOSIP.664 trimer was not affected by preincubation of up to 10 days at 37 °C, indicating the exceptional robustness of the disulfide mutant BG505 trimers (Fig. S4). The binding site for gl-PG9 also remained stable over time on the V1-V2 disulfide mutant trimers, although incubation at 37 °C reduced the binding strength by 40% (Fig. S4).

**Figure 5.**
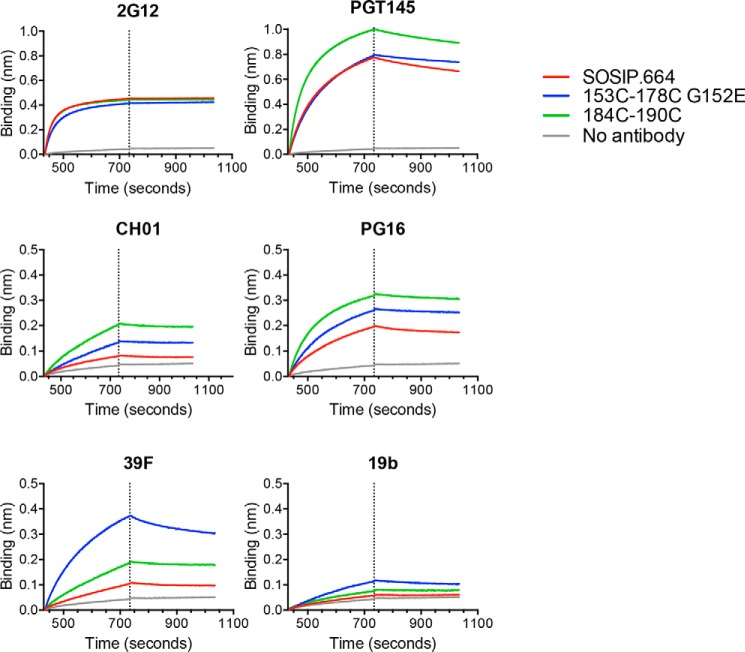
**Antigenicity of BG505 SOSIP.664 disulfide mutants.** Biolayer interferometry sensorgrams show that the binding of the indicated bNAbs and non-NAbs to various PGT145-purified trimers (80 nm). The *dotted lines* indicate the end of the association phase (duration, 300 s) and the start of the dissociation phase (duration, 300 s). The experiments were performed twice independently, and one representative experiment is shown.

To study the Ab–antigen interaction in a context that better resembles the process of a B-cell receptor encountering an antigen, we also analyzed the antigenicity of the trimers by biolayer interferometry. Here, bNAbs are coated to the protein A sensor followed by incubation of soluble purified BG505 trimers. In line with the results in ELISA, binding of V2 bNAbs PGT145, CH01, and PG16 to V1-V2 disulfide mutant trimers was enhanced compared with binding to SOSIP.664 (Fig. 5). The V2-internal disulfide bond mutant trimer showed the largest increase in V2 bNAb binding while showing a minor increase in binding to V3 non-NAbs 19b and 39F (Fig. 5).

### Improved neutralization of V1-V2 and V2-internal disulfide bond virus mutants by V2 bNAbs

**Figure 6.**
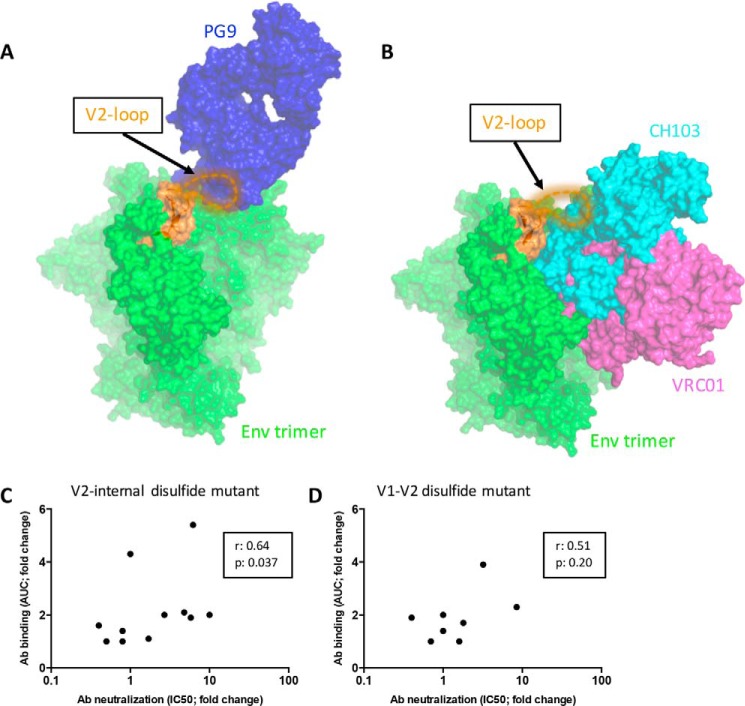
**Stabilizing the flexible V2 loop improves recognition of the BG505 Env trimer by the V2-apex bNAbs and some CD4bs bNAbs.** *A*, superposition of the crystal structure of the BG505 SOSIP.664 trimer (*green*; PDB code 5CEZ) and the PG9 antibody (*blue*; PDB code 3U4E) in complex with a scaffolded V1V2 domain (26, 44). The nine amino acids (ENQGNRSNN) in the V2 loop for which density is lacking in the trimer crystal structure are depicted as an *orange dotted line. B*, a similar superposition of the same trimer structure and the VRC01 in complex with gp120 (*purple*; PDB code 3NGB) and CH103 (*cyan*; PDB code 4JAN) in complex with gp120 (81, 86). As in *A*, an *orange dotted line* depicts the residues in the V2 loop that lack density in the crystal structure. *C* and *D*, correlation plots between bNAb binding efficiency (fold change in AUC compared with BG505 SOSIP.664 trimer) and bNAb neutralization capacity (fold change in IC_50_ compared with BG505.T332N virus. *C*, I184C/E190C V2-internal disulfide mutant. *D*, E153C/R178C/G152E V1-V2 disulfide mutant. The *r* and *p* values for nonparametric Spearman correlations are shown in the *boxes*.

To determine whether the engineered disulfide bonds increased the presentation of V2 bNAb and gl-bNAb epitopes on the native, virion-associated trimer, we made variants of the BG505.T332N Env-pseudotyped virus containing the E153C/R178C/G152E or I184C/E190C changes. The V2-internal disulfide mutant virus was more sensitive to neutralization by V2 bNAbs compared with BG505 (by 5-fold for PG9, 3-fold for PG16, 6-fold for CH01, 3-fold for PGDM1400, and 6-fold for gl-PG16), although there was no difference in the sensitivity of the two viruses to PGT145 and VRC26.09, and gl-PG9 neutralized neither virus (Fig. S5 and Table 3). For the V1-V2 disulfide mutant virus, the only difference compared with the WT virus was observed with gl-PG16, which was 3-fold more potent (Fig. S5 and Table 3). Neutralization sensitivity to antibodies 2G12 and PGT151 was similar between BG505.T332N and disulfide mutant viruses, indicating that the overall NAb sensitivity was not affected (Fig. S5). The data pattern implies that the V2 bNAb and gl-bNAb epitopes are not stabilized in the same way by the two disulfide mutational strategies tested here. Superposition of the Env trimer structure with a V1V2 scaffold bound by PG9 shows that the V2 loop can interfere with V2 bNAb binding (Fig. 6*A*). Whether the V1-V2 disulfide bond mutant improves binding of V2 bNAbs to the BG505 Env trimer obviously depends on the position of the stabilized V2 loop and might be different for the V1-V2 disulfide and V2-internal disulfide mutant trimers.

**Table 3:**
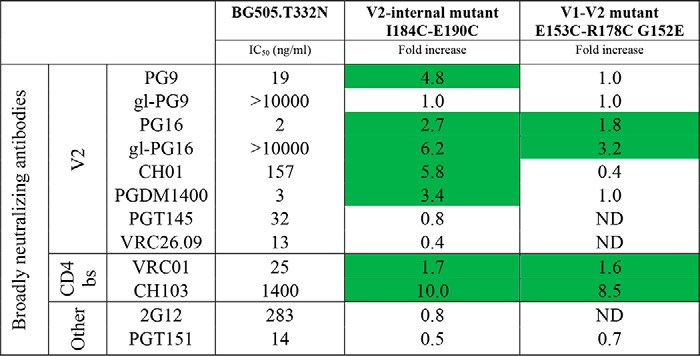
**Neutralization sensitivity of BG505.T332N virus disulfide mutants** Neutralization sensitivity of the BG505.T332N Env-pseudotyped virus and disulfide mutant stabilized variants. The IC_50_ values for the listed bNAbs and gl-bNAbs were calculated from the neutralization curves for BG505.T332N (Fig. S5). The corresponding values for the mutant viruses are presented as fold increases compared to BG505.T332N. An increase in neutralization sensitivity (>1.5 fold) is highlighted in green. ND, not determined.

We noted that both disulfide bond mutant viruses were ∼10-fold more sensitive to neutralization by CH103, whereas there was no difference in sensitivity to VRC01, which also recognizes a CD4bs epitope (Fig. S5). Whether the V2 loop interferes with how different bNAbs see their CD4bs epitopes is likely to be influenced by their angle of approach to the trimer. Because CH103 approaches at a 45° angle relative to the trimer axis, it comes into close proximity with the V2 region; hence any alteration in the orientation of V2 caused by an engineered disulfide could alter access of CH103 to its epitope (Fig. 6*B*). In contrast, VRC01 approaches the CD4bs at an angle parallel to the virus membrane and at 90° to the trimer axis, which means that its neutralization potency is less likely to be affected by the precise orientation of the V2 loop (Fig. 6*B*).

Overall, the virus neutralization data and SOSIP.664 trimer-binding data were generally concordant. Compared with their unmodified counterparts, the V1-V2 and V2-internal disulfide bond mutant trimers generally interact more strongly with V2 bNAbs and gl-bNAbs compared with SOSIP.664 trimers, and this is also true for neutralization of the corresponding Env-pseudotyped viruses. Thus, increases in Ab binding to the V2-internal disulfide bond mutant trimer and neutralization of the corresponding mutant virus by the same Ab were correlated (*r* = 0.64, *p* = 0.037; Fig. 6*C*). However, there were also examples, such as gl-PG9, where stronger trimer binding did not translate into more potent neutralization. The correlation was not statistically significant for the V1-V2 disulfide bond mutant trimer and virus (*r* = 0.51, *p* = 0.20; Fig. 6*D*). Thus, stabilization of the V2 domain either by a disulfide bond within the V2 loop or by one that links the V1 and V2 domains can improve the presentation of V2 bNAb and/or gl-bNAb epitopes at the trimer apex. However, the beneficial effects of the engineered disulfide bond(s) are not universal for all the V2-targeted antibodies, and the two strategies do not lead to identical outcomes.

## Discussion

SOSIP trimers have become a widely used platform for structure-guided immunogen design strategies that aim to elicit bNAbs in any of various possible ways. Here, we sought to improve their presentation of bNAb and/or gl-bNAb epitopes, in particular those located at the trimer apex, by reducing local conformational flexibility. To do so, we designed and characterized V1-V2 and V2-internal disulfide mutants of the BG505 SOSIP.664 trimer that were intended to stabilize the V2 loop and its associated bNAb epitopes. Three steps were required to create the desired disulfide mutant trimers: structure-based design, virus evolution, and positive selection. We initially designed five mutants with appropriately positioned cysteine pairs (*i.e.* at residues that were close enough to allow formation of a disulfide bond), but only two of them (E153C/R178C and I184C/E190C) yielded trimers with reasonable efficiency. Even for these mutants, there was a marked reduction in trimer yield compared with the SOSIP.664 trimer. We assume that the introduced cysteine residues interfere with the intricate process of gp120 folding and trimer assembly, perhaps by forming aberrant disulfide bonds leading to dead-end folding pathways or by distorting the gp120 subunit enough to impede trimer formation (11, 19–21, 59, 67).

The E153C/R178C V1-V2 disulfide bond mutant formed trimers particularly poorly, and when trimers did form, they frequently sampled unusually open configurations that resembled the CD4-bound conformation of the SOSIP.664 trimer. Any adverse effects of the introduced cysteine residues may be exacerbated when they are in different domains. We therefore implemented a second procedure, virus evolution, to see whether some of these defects could be overcome.

We have previously shown that virus evolution is an effective technique for repairing folding problems associated with modifications in the V1V2 domain and/or changes in the disulfide bond architecture of Env proteins (59–62). Here, we used this method to drive the evolution of compensatory mutations that improved the stability, yield, and antigenicity of a replication impaired E153C/K178C V1-V2 disulfide mutant LAI virus. The most substantive improvement to virus replication was conferred by the G152E change that is positioned directly adjacent to the V1-component of the interdomain disulfide bond. We do not yet know how the G152E compensatory mutation acts, but it may increase the propensity of the adjacent cysteine 153 residue to pair successfully with its V2 counterpart, cysteine 178. Alternatively, it may stabilize the conformation of the V1V2 domain once the interloop disulfide bond is formed. When the G152E substitution was made in the E153C/R178C V1-V2 disulfide bond mutant SOSIP.664 trimer, its conformational stability was improved, and its propensity to adopt the unusually open conformation was reduced. Hence, what was learned from the virus evolution method was translatable to the context of the SOSIP.664 trimer.

An additional change that also emerged in the evolution culture, S128R, conferred a modest additional improvement to infectivity. We note that a change at residue 128 (S128N) arose in an evolutionary rescue culture of an HxB2 virus clone that was crippled by the deletion of a key glycan at position 262. In that case, the S128N change compensated for a substantial folding defect in the gp120 subunit (68–70). We have not further explored how the S128R change affects the SOSIP.664 trimer.

The third step that was required to isolate native-like disulfide mutant trimers was affinity purification using the trimer-specific PGT145 bNAb. This positive-selection procedure allowed the isolation of stable, native-like V1-V2 and V2-internal disulfide mutant trimers. Nevertheless, the eventual yields of the V1-V2 and V2-internal disulfide trimers described here were still substantially (4- and 3-fold, respectively) lower than that of the parental BG505 SOSIP.664 trimer. If these mutant trimers prove to be useful components of a bNAb-induction strategy, additional steps will need to be taken to address this limitation. For example, we have found that expressing a mutant trimer in a stable CHO cell line can sometimes improve the yield by severalfold, and modifying key residues at the gp120-gp41 interface can also be beneficial.

The V2-internal disulfide bond did improve the trimer binding of V2 bNAbs. One explanation may be that the flexible V2 loop assumes a conformation in which V2 bNAb epitopes are transiently or permanently occluded on a subpopulation of BG505 SOSIP.664 trimers. Such masking could be mediated by the protein or the glycan components of the V2 loop itself or of the nearby V1 loop. Whatever the explanation, several V2 and one CD4bs bNAb epitopes were better presented on the V2-internal disulfide mutant trimer, perhaps because the additional cross-link stabilizes the loop and reduces its propensity to adopt a conformation in which certain epitopes are at least partially occluded. The disulfide bond connecting the V1 and V2 loops also improved the presentation of V2 bNAb epitopes, although less so than the V2-internal disulfide. We also intend to determine whether the V2-internal and V1-V2 disulfides can be incorporated into SOSIP trimers of other genotypes. The length of the V2 loop may be a variable that affects, in particular, the stabilizing and/or other effects of the V2-internal disulfide. Indeed, removal of 7 amino acids from the BG505 V2 contributed to the engagement of V2 gl-bNAbs (71).

For an HIV-1 Env immunogen to induce bNAbs, it is likely to be necessary that it can first activate naïve B-cells that express appropriate gl-bNAb epitopes as their BCRs. A complex evolutionary process may then drive the emergence of a true bNAb. In this context, we found that the affinity of three inferred V2 gl-bNAb precursors for the V2-internal disulfide mutant was increased compared with SOSIP.664. Such an affinity increase may enhance the probability that naïve B-cells expressing similar gl-NAb epitopes will be found and activated when the modified trimers are used as immunogens. Whether improved antigenicity of the V2-internal disulfide mutant trimer correlates with an improved immunogenicity has to be addressed in future immunization studies. Such studies remain to be initiated, for example using knockin mice that express V2 gl-bNAbs as BCRs (72, 73). The disulfide stabilization approach described here might be complementary to other approaches aimed at improving recognition by V2-apex gl-bNAbs (71, 74).

A V2-internal disulfide mutant (Cys^183^–Cys^191^) of the clade C ZM109F Env-pseudotyped virus has recently been described (75). The location of that disulfide is similar to the one we describe here, *i.e.* I184C/E190C. In the context of the ZM109F virus, the disulfide improved neutralization by Abs that bind the integrin α4β7-binding LD(V/I) motif in V2. However, in contrast to the effect of the disulfide that we introduced into the BG505.T332N virus, the ZM109F mutant virus was not more sensitive to neutralization by bNAb PG9 (75). The effect of a V2-internal disulfide may therefore be genotype-dependent.

From an evolutionary perspective, it might be interesting to determine what selective pressure was driving the evolutionary loss of this twin cysteine motif in HIV-1 and when. After evolving in several SIV lineages, the twin cysteine motif disappeared from most, but not all SIVcpz isolates and was then lost completely in HIV-1 (18). The presence of this motif, which restrains the flexibility of the V2 loop, might have favored the induction of V2 NAbs that exerted selection pressure. Indeed several V1V2 targeting SIV NAbs have been isolated, providing evidence for the presence of such a selective pressure on the V1V2 loop structure (76). Thus, we can speculate that the evolution from SIV to HIV might have involved enhancing the flexibility of the V1 and V2 loops to prevent productive interactions with naïve B-cells with the capacity to evolve into V2-targeting (b)NAbs.

In conclusion, we designed and characterized V2-internal and V1-V2 disulfide mutants to stabilize the V1 and/or V2 variable loops on the BG505 SOSIP.664 trimer. The presentation of several V2 and in some cases also CD4bs (gl-)bNAb epitopes was improved when the V1 and V2 loops were stabilized by the V1-V2 disulfide and more so by the V2-internal disulfide.

## Materials and methods

### Construct design

The BG505 SOSIP.664 construct has been described elsewhere (42). It was generated by introducing the following sequence changes: A501C and T605C (gp120-gp41ECTO disulfide bond) (77); I559P in gp41ECTO (trimer-stabilizing) (78); REKR to RRRRRR in gp120 (cleavage enhancement) (79); and a stop codon at gp41_ECTO_ residue 664 (improvement of homogeneity and solubility) (80). SOSIP.664-D7324 trimers contain a D7324 epitope-tag sequence at the C terminus of gp41_ECTO_ and were constructed by adding the amino acid sequence GSAPTKAKRRVVQREKR after residue 664 in gp41_ECTO_ (42). Point mutants were generated by QuikChange site-directed mutagenesis (Agilent, Stratagene) and verified by sequencing. All experiments described in this manuscript used D7324-tagged trimers.

### Env protein expression

The various SOSIP Env constructs were expressed by polyethyleneimine-mediated transient transfection (together with the furin gene) of adherent HEK 293T cells or of HEK 293F cells adapted for serum-free suspension cultures, as previously described (42, 47). For small-scale trimer expression, 293T cells were used, and for larger-scale trimer production, 293F cells were used. All experiments were performed with purified trimers utilized 293F cell-expressed proteins.

### Trimer purification

For screening purposes, unpurified 293T-expressed trimers were analyzed directly using the culture supernatants obtained 6 days post-transfection. For extensive analysis of V2 bNAb reactivity, Env trimers were purified from 293F transfection supernatants by affinity chromatography using a PGT145 column or a 2G12 column followed by SEC, as described (42, 47, 63). Protein concentrations were determined using UV280 absorbance, and theoretical extinction coefficients were obtained via the ExPASy webserver (ProtParam tool). Except for the experiments described in Fig. 3*B*, which included 2G12-purified trimers, all other experiments were performed with PGT145-purified trimers.

### SDS-PAGE and BN-PAGE

Env proteins were analyzed using SDS-PAGE and BN-PAGE, followed by Western blotting or Coomassie Blue dye staining as previously described (47, 78).

### D7324-capture ELISA

The method to perform sandwich ELISAs using D7324-tagged BG505 SOSIP.664 trimers has been described in detail elsewhere (42, 82). In summary, trimers were immobilized at 1.0 μg/ml via their D7324 tags for 2 h at room temperature on half-well 96-well plates (Greiner) precoated with Ab D7324 (Aalto Bioreagents) at 10 μg/ml in 0.1 m NaHCO_3_ (pH 8.6) overnight. Serially diluted Abs were added for 2 h, washed, and then detected using horseradish peroxidase–labeled goat anti-human immunoglobulin (Jackson Immunoresearch). We also used a modified D7324-capture ELISA protocol that was optimized for detecting gl-bNAb binding (66). The modifications compared with the regular protocol are as follows. Purified BG505 SOSIP.664-D7324 trimers were added at 5 μg/ml instead of 2 μg/ml, and casein-blocking buffer (Thermo Scientific) was used as blocking agent instead of 2% milk in TBS.

### Lectin ELISA

Purified BG505 trimers were incubated in PBS at room temperature or at 37 °C for 0, 60, 120, 180, or 240 h, and subsequently antigenicity was assessed with a lectin ELISA. Greiner half-well ELISA plates were coated with *Galanthus nivalis* lectin (Vector Laboratories), followed by blocking of the plates with casein blocking buffer. Then purified trimers were transferred to the plates in a concentration of 2.5 μg/ml and incubated at room temperature for 2 h. Next, binding of several antibodies to the BG505 trimers was tested using the following Ab concentrations: 2G12, 0.75 μg/ml; PGT145, 0.75 μg/ml; glPG9, 5.0 μg/ml; glPG16, 5.0 μg/ml; glCH01, 5.0 μg/ml; PG9, 0.75 μg/ml; PG16, 0.75 μg/ml; and CH01, 0.75 μg/ml. After incubating the antibodies for 1 h, similar steps were followed as for the D7324 ELISA to detect antibody binding.

### Biolayer interferometry

Antibody binding to purified BG505 trimers was studied using a ForteBio Octet K2. All assays were performed at 30 °C and with agitation set at 1000 rpm. Trimer and antibody dilutions were made in running buffer (PBS, 0.1% BSA, 0.02% Tween 20) in a final volume of 200 μl/well. Antibody was loaded on protein A sensors (ForteBio) at 1.0 μg/ml in running buffer until a binding threshold of 0.5 nm was reached. Trimers were diluted in running buffer at 80 nm, and association and dissociation were measured for 300 s. Trimer binding to a protein A sensor not loaded by antibody was set as background.

### Negative-stain electron microscopy (EM)

PGT145-purified BG505 trimers were analyzed by negative stain EM as previously described (47, 63, 83).

### Differential scanning calorimetry

Thermal denaturation of purified Env proteins was studied using a MicroCal VP-Capillary DSC calorimeter (Malvern Instruments) or a nano-DSC calorimeter (TA Instruments) as described previously (47). All Env protein samples were first extensively dialyzed against PBS, and the protein concentration was then adjusted to 0.1–0.3 mg/ml. After loading the sample into the cell, thermal denaturation was probed at a scan rate of 60 °C/h. Buffer correction, normalization, and baseline subtraction procedures were applied before the data were analyzed using NanoAnalyze software version 3.3.0 (TA Instruments) or Origin 7.0 software. The data were fitted using a two-state model.

### Disulfide bond analysis of BG505 trimers

The disulfide bond patterns of SOSIP.664 trimer variants were determined as described previously (19, 20). In brief, samples containing 20 μg of trimer were alkylated with a 10-fold molar excess of 4-vinylpyridine for 1 h at room temperature in the dark, to cap free cysteine residues. Deglycosylation was performed by incubating trimers with 1 μl of peptide:*N*-glycosidase F solution (500,000 units/ml) in 100 mm ammonium citrate buffer (pH 6.5) for 1 week at 37 °C. The fully deglycosylated, and alkylated samples were digested overnight with trypsin (protein to enzyme ratio of 30:1) at 37 °C and were then analyzed by LC-MS using an Orbitrap Velos Pro^TM^ hybrid (Thermo Scientific, San Jose, CA) mass spectrometer equipped with electron transfer dissociation module and coupled to an Acquity ultra performance liquid chromatography system (Waters, Milford, MA). Approximately 5 μl (1 μg equivalent) of the tryptic digest was injected onto a C18 PepMap^TM^ 300 column (300-μm inner diameter × 15 cm, 300 Å; Thermo Scientific, Sunnyvale, CA), and the peptides were separated using a linear gradient starting from 3% B to 40% B gradient in 50 min and then 90% B for 10 min followed by re-equilibration at 97% A for 10 min. LC-MS runs were performed with a flow rate of 5 μl/min using mobile phases consisting of solvent A: 99.9% HPLC-grade H_2_O + 0.1% formic acid and solvent B: 99.9% HPLC-grade CH3CN + 0.1% formic acid. Data were collected using the data-dependent mode. To determine the disulfide connectivity profiles, the five most intense ions in a high-resolution scan in the Orbitrap were subjected to alternating collision-induced dissociation and electron transfer dissociation in the linear ion trap. The resulting data were analyzed using the Mascot search engine to identify peptides containing free cysteine residues, and the disulfide bond patterns were then determined manually, as described previously (19, 20).

### HIV-1 LAI Env-pseudotyped virus mutants

The pRS1 plasmid expressing the full-length LAI molecular clone (pLAI) was the basis for the WT and mutant viruses used in these studies (84). *Env* genes were mutated in the pRS1 plasmid using the QuikChange mutagenesis kit (Stratagene) and cloned into pLAI as SalI–BamHI fragments. The integrity of all plasmids was verified by sequencing. To create a PG9-sensitive LAI variant, four substitutions (S162T, G167D, V169K, and E172V) were introduced into β-strand C of the V1V2 domain; the substitutions were based on the corresponding residues in the BG505 isolate, which is sensitive to PG9 (42). The outcome was a 20-fold increase in sensitivity to PG9 neutralization (IC_50_ = 0.1 μg/ml compared with 2.0 μg/ml for the parental LAI virus). The resulting LAI-PG9 virus was then used as the basis for the disulfide mutant viruses described in the results.

### Virus evolution cultures

Evolution experiments were performed as described before (14, 59, 61). A total of 5 × 10^6^ SupT1 cells were transfected with 5 or 20 μg of LAI-PG9. Virus replication in the cultures was monitored twice a week by visual inspection for the appearance of syncytia and by using a p24-antigen ELISA. The cultures were maintained for 3 months, during which period cell-free supernatants were passaged onto uninfected cells when virus replication was apparent. Smaller quantities of supernatant were used for passaging when the extent of virus replication was sufficient to cause significant levels of cell destruction in the cultures. At regular intervals, cells and filtered supernatant were collected for storage at −80 °C until needed for genotypic and phenotypic analyses. When a substantial increase in virus replication was identified, DNA was extracted from the infected cells using the QIAamp DNA mini kit (Qiagen). The complete proviral *env* genes were PCR-amplified using primers 1 (5′-ATAAGCTTAGCAGAAGACAGTGGCAATG-3′) and 2 (5′-GCAAAATCCTTTCCAAGCCC-3′) and then sequenced.

### Single-round infection assay and neutralization assay

LAI viruses and BG505 Env-pseudotyped viruses were generated by transfection of 293T cells as described previously (14, 47, 59). Concentration of virus particles in the supernatant was determined with a p24-antigen ELISA. The TZM-bl reporter cell line, which stably expresses high levels of CD4 and the co-receptors CCR5 and CXCR4 and contains the luciferase and β-gal genes under the control of the HIV-1 long-terminal-repeat promoter, was obtained through the National Institutes of Health AIDS Research and Reference Reagent Program, Division of AIDS, NIAID, National Institutes of Health (John C. Kappes, Xiaoyun Wu, and Tranzyme Inc., Durham, NC). To determine infectivity of LAI-PG9 WT and mutant viruses as well as virus evolution supernatant, a fixed amount of virus (1 ng of CA-p24-antigen equivalent) was tested on the TZM-bl reporter cell line as described previously (47, 85). To determine neutralization capacity of monoclonal antibodies, a fixed amount of BG505 Env-pseudotyped virus (1 ng of CA-p24-antigen equivalent) was incubated for 30 min at room temperature with 3-fold serial dilutions of the antibody. All infection and neutralization assays were performed in duplicate. Uninfected cells were used to correct for background luciferase activity. The infectivity of each virus in the absence of antibody was set at 100%. Nonlinear regression curves were determined, and the 50% inhibitory concentration (IC_50_) was calculated using a sigmoid function in Prism software version 5.0.

## Author contributions

S. W. d. T., I. A. W., J. P. M., and R. W. S. conceptualization; S. W. d. T., M. M.-R., and R. W. S. resources; S. W. d. T., E. P. G., K. S., A. T. d. l. P., and K. B. data curation; S. W. d. T., E. P. G., K. S., A. T. d. l. P., K. B., M. M.-R., W.-H. L., H. D., and R. W. S. formal analysis; S. W. d. T. and R. W. S. investigation; S. W. d. T. visualization; S. W. d. T. methodology; S. W. d. T. and R. W. S. writing-original draft; S. W. d. T. and R. W. S. project administration; S. W. d. T., K. S., H. D., I. A. W., J. P. M., A.B.W., and R. W. S. writing-review and editing; H. D., J. P. M., A.B.W., and R. W. S. supervision; J. P. M., A.B.W., and R. W. S. funding acquisition; A.B.W. software; R. W. S. validation.

## Supplementary Material

Supporting Information

## Footnotes

2 The abbreviations used are:

bNAb: broadly neutralizing antibody
NAb: neutralizing antibody
gl: germline
NS: negative stain
AUC: area under the curve
BCR: B-cell receptor
PDB: Protein Data Bank
BN: Blue Native
SEC: size-exclusion chromatography
DSC: differential scanning calorimetry.
