# Supplementary material for: Stabilization of the V2 loop improves the presentation of V2 loop–associated broadly neutralizing antibody epitopes on HIV-1 envelope trimers
Source: J Biol Chem. 2019 Feb 6;294(14):5616–31. doi: 10.1074/jbc.RA118.005396 (PMC6462529; doi:10.1074/jbc.RA118.005396)
Supplement: Supporting Information [file supp_294_14_5616__index.html]

Stabilization of the V2 loop improves the presentation of V2 loop–associated broadly neutralizing antibody epitopes on HIV-1 envelope trimers — Stabilizing the V2 loop of HIV-1 envelope trimers — Stabilization of the V2 loop improves the presentation of V2 loop–associated broadly neutralizing antibody epitopes on HIV-1 envelope trimers — Stabilizing the V2 loop of HIV-1 envelope trimers — Supporting Information 

# Stabilization of the V2 loop improves the presentation of V2 loop–associated broadly neutralizing antibody epitopes on HIV-1 envelope trimers

## Supporting Information

- Supporting Information (to be published online) - Figure legends of supplementary figures
