# Supplementary material for: Stabilization of the V2 loop improves the presentation of V2 loop–associated broadly neutralizing antibody epitopes on HIV-1 envelope trimers
Source: J Biol Chem. 2019 Feb 6;294(14):5616–31. doi: 10.1074/jbc.RA118.005396 (PMC6462529; doi:10.1074/jbc.RA118.005396)
Supplement: Supporting Information [file supp_RA118.005396_140159_1_supp_281562_pmxb2v.pdf]

SI Figures

SI Figure 1. Expression and Ab binding of unpurified BG505 SOSIP.664 disulfide bond mutants. (**A**) The indicated unpurified wild type and V1-V2 (left panel) or V2-internal (right panel) disulfide mutant SOSIP.664 proteins, present in culture supernatants from transfected 293T cells, were analyzed by SDS-PAGE followed by western blotting with MAb ARP3119. (**B, C**) Binding of the indicated bNAbs to the same unpurified Env proteins, assessed by D7324-capture ELISA. (**B**) Wild type and V1-V2 disulfide mutant SOSIP.664 proteins. (**C**) Wild type and V2-internal disulfide mutant SOSIP.664 proteins.

SI Figure 2. Repair of the E153C-R178C V1-V2 disulfide bond mutant
(**A**) Unpurified wild type and E153C-R178C V1-V2 disulfide bond mutant SOSIP.664 proteins, present in culture supernatants from transfected 293T cells, were analyzed by SDS-PAGE followed by western blotting with MAb ARP3119. The V1-V2 disulfide mutants contain or lack a compensatory change at residue-152, as indicated, (**B**) Binding of the indicated bNAbs to the same unpurified Env proteins, assessed by D7324-capture ELISA.

SI Figure 3. Disulfide-bond analysis of BG505 SOSIP.664 disulfide mutants
(**A**) Overview and characterization of all individual disulfide-linked peptides that were identified by mass spectrometry in the disulfide-bond analysis of PGT145-purified wild type and disulfide SOSIP.664 mutant trimers. The disulfide-linked peptides corresponding to the engineered V1-V2 and V2-internal disulfide bonds are highlighted in orange (**B**) The indicated aberrant disulfide-linked peptides were identified, at low abundance (<5%), in wild type and/or disulfide mutant SOSIP.664 trimers.

SI Figure 4. Stability of V1V2 bNAb epitope on BG505 SOSIP.664 disulfide mutants over time
Binding of V1V2 bNAbs to purified BG505 trimers that were pre-incubated at RT or 37°C for 0, 60, 120, 180 or 240 hours, as determined by lectin ELISA. Antibody binding to BG505 trimers at t=0 was used as a reference and was set to 100% binding. Gl-PG16 en gl-CH01 did not bind to any of the trimers at t=0.

SI Figure 5. Neutralization sensitivity of BG505.T332N disulfide mutant viruses
Neutralization of the wild type and V1-V2 or V2-internal disulfide mutant BG505.T332N viruses by the indicated bNAbs or gl-bNAbs was measured in a TZM-bl cell assay. Note that the concentration ranges on the x-axis differ between antibodies. Infectivity in the absence of an antibody was defined as 100%. Neutralization sensitivity of the disulfide mutant viruses was tested in duplo in two individual experiments (n=4). Mean IC_{50} values are listed in table 3.

SI Figure 1

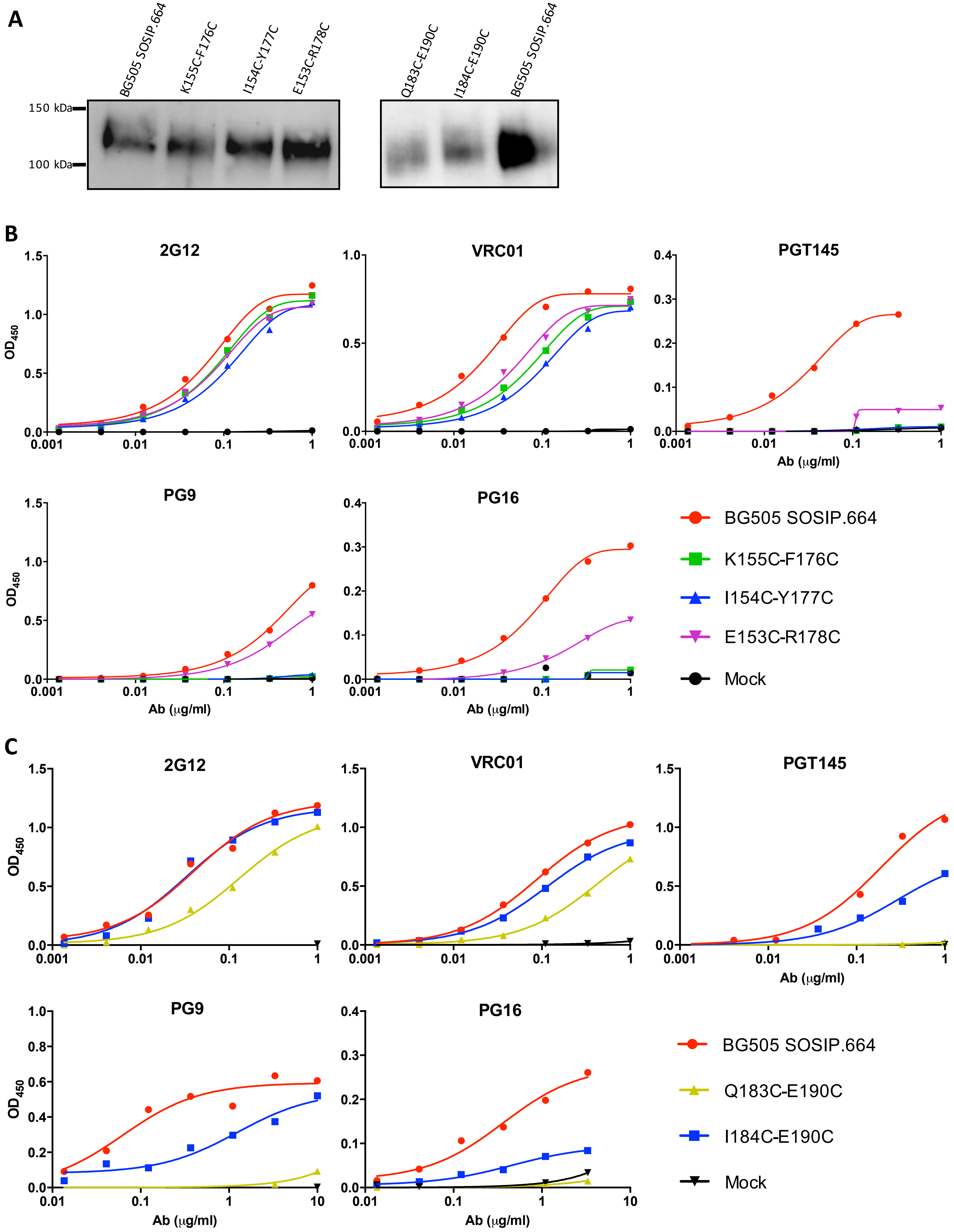

A
BG505 SOSIP.664
K155C-F176C
I154C-Y177C
E153C-R178C
Q183C-E190C
I184C-E190C
BG505 SOSIP.664
150 kDa
100 kDa
B
2G12
VRC01
PGT145
PG9
PG16
OD_{450}
Ab (μg/ml)
BG505 SOSIP.664
K155C-F176C
I154C-Y177C
E153C-R178C
Mock
C
2G12
VRC01
PGT145
PG9
PG16
OD_{450}
Ab (μg/ml)
BG505 SOSIP.664
Q183C-E190C
I184C-E190C
Mock

SI Figure 2

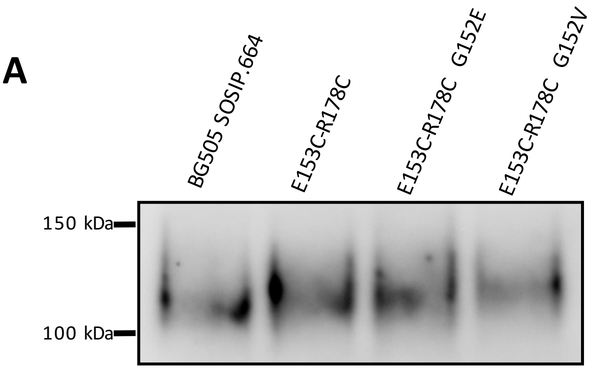
A
BG505 SOSIP.664
E153C-R178C
E153C-R178C G152E
E153C-R178C G152V
150 kDa
100 kDa

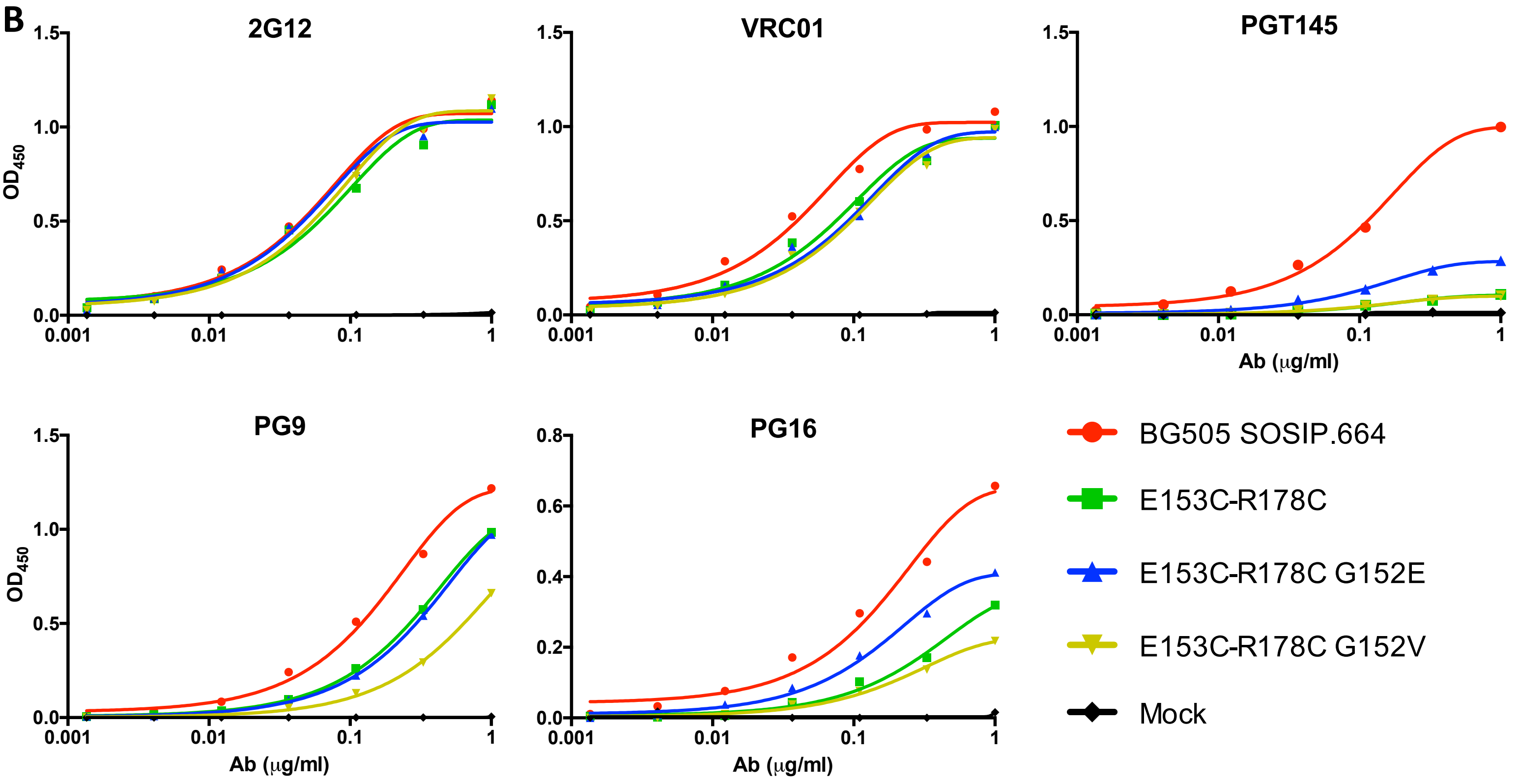
B
2G12
VRC01
PGT145
PG9
PG16
OD450
Ab (μg/ml)
BG505 SOSIP.664
E153C-R178C
E153C-R178C G152E
E153C-R178C G152V
Mock

SI Figure 3

A #1: BG505 SOSIP.664

Disulfide Loop Domain	Disulfide-Linked Peptides	BG505 SOSIP.664 D7324 Tag (#1)				
		CS	Theoretical *m/z*	Experimental *m/z*	Mass Error (ppm)	MS/MS
I	DAETTLFC^{54}ASDAK<br>HNVWATHAC^{74}VPTDPNPQEIHLED^{88}VTEEFNMWK	3+	1719.4427	1719.4546	7	
		4+	1289.8338	1289.8408	5	
		5+	1032.0685	1032.0738	5	✓
		6+	860.2250	860.2305	6	
		7+	737.4796	737.4849	7	
II	NNMVEQMHTDIISLWDQSLKPC^{119}VK<br>LINC^{196}D^{197}TSAITQAC^{205}PK<br>LTPLC^{126}VTLQC^{131}TD^{132}VTND^{136}ITDDMR<br>D^{156}C^{157}SFD^{160}MTTELR	7+	1169.8301	1169.8396	8	
		8+	1023.7272	1023.7354	8	✓
		9+	910.0917	910.0988	8	
		10+	819.1832	819.1838	1	
III	C^{228}K<br>FN^{234}GTGPC^{239}PSVSTVQC^{247}THGIKPVVSTQLLLN^{262}GSLAEEEVMIR<br>VSFEPIPIHYC^{218}APAGFAILK	5+	1346.6868	1346.6850	1	
		6+	1122.4069	1122.4081	1	
		7+	962.2069	962.2084	2	✓
		8+	842.0570	842.0582	1	
IV	NILVQFNTPVQID^{295}C^{296}TRPND^{301}NTR<br>QAHC^{331}D^{332}VSK	2+	1721.8332	1721.8397	4	
		3+	1148.2245	1148.2222	7	
		4+	861.4202	861.4263	7	✓
		5+	689.3376	689.3422	7	
		6+	574.6159	574.6192	6	
V	FADSSGGDLEVTTHSFNC^{378}GGEFFYC^{385}DTSGLFDSTWISDTSVQGSDSTGSDDSITLPC^{418}R<br>C^{445}VSDITGLILTR	4+	1852.5669	1852.5776	6	
		5+	1482.2549	1482.2664	8	✓
		6+	1235.3803	1235.3905	8	
gp41 SOSIP	C^{501}K<br>LIC^{604}C^{605}TNVPWN^{611}SSWSNR<br>DQQLLGIWGC^{598}SGK	3+	1176.8808	1176.8876	6	
		4+	882.9124	882.9182	7	✓
		5+	706.5314	706.5362	7	
		6+	588.9440	588.9479	5	

#2: E153C-R178C

Disulfide Loop Domain	Disulfide-Linked Peptides	BG505 SOSIP.664 E153C-R178C D7324 Tag (#2)				
		CS	Theoretical *m/z*	Experimental *m/z*	Mass Error (ppm)	MS/MS
I	DAETTLFC^{54}ASDAK<br>HNVWATHAC^{74}VPTDPNPQEIHLED^{88}VTEEFNMWK	3+	1719.4427	1719.4439	1	
		4+	1289.8338	1289.8371	3	
		5+	1032.0685	1032.0728	4	✓
		6+	860.2250	860.2299	6	
		7+	737.4796	737.4818	3	
II	NNMVEQMHTDIISLWDQSLKPC^{119}VK<br>LINC^{196}D^{197}TSAITQAC^{205}PK<br>LTPLC^{126}VTLQC^{131}TD^{132}VTND^{136}ITDDMR<br>D^{156}C^{157}SFD^{160}MTTELR	7+	1169.8301	1169.8345	4	
		8+	1023.7272	1023.7321	5	✓
		9+	910.0917	910.0959	5	
		10+	819.1832	819.1879	6	
	GC^{153}LK<br>VYSLFYC^{178}LDVVQINENQGNR	2+	1396.1807	1396.1830	2	
		3+	931.1229	931.1261	3	✓
		4+	698.5940	698.5987	7	
		5+	559.0766	559.0776	2	
III	C^{228}K<br>FD^{234}GTGPC^{239}PSVSTVQC^{247}THGIKPVVSTQLLLD^{262}GSLAEEEVMIR<br>VSFEPIPIHYC^{218}APAGFAILK	5+	1347.0804	1347.0847	3	
		6+	1122.7349	1122.7391	4	
		7+	962.4881	962.4933	5	✓
		8+	842.3030	842.3072	5	
		9+	748.8257	748.8284	4	
IV	NILVQFNTPVQID^{295}C^{296}TRPND^{301}NTR<br>QAHC^{331}D^{332}VSK	2+	1721.8332	1721.8408	4	
		3+	1148.2245	1148.2288	4	
		4+	861.4202	861.4243	5	✓
		5+	689.3376	689.3419	6	
		6+	574.6159	574.6182	4	
V	FADSSGGDLEVTTHSFNC^{378}GGEFFYC^{385}DTSGLFDSTWISDTSVQGSDSTGSDDSITLPC^{418}R<br>C^{445}VSDITGLILTR	4+	1852.5669	1852.5657	1	
		5+	1482.2549	1482.2575	2	✓
		6+	1235.3803	1235.3801	0.6	
gp41 SOSIP	C^{501}K<br>LIC^{604}C^{605}TNVPWN^{611}SSWSNR<br>DQQLLGIWGC^{598}SGK	3+	1176.8808	1176.8837	3	
		4+	882.9124	882.9147	3	✓
		5+	706.5314	706.5331	3	
		6+	588.9440	588.9447	1	

SI Figure 3

A #3: E153C-R178C G152E

Disulfide Loop Domain	Disulfide-Linked Peptides	BG505 SOSIP.664 G152E-E153C-R178C D7324 Tag (#3) CS	Theoretical *m/z*	Experimental *m/z*	Mass Error (ppm)	MS/MS
I	DAETTLFC^{54}ASDAK<br>HNVWATHAC^{74}VPTDPNPQEIHLED^{88}VTEEFNMWK	3+	1719.4427	1719.4430	0.2	✓
		4+	1289.8338	1289.8363	2	
		5+	1032.0685	1032.0706	2	
		6+	860.2250	860.2289	5	
		7+	737.4796	737.4811	2	
II	NNMVEQMHTDIISLWDQSLKPC^{119}VK<br>LINC^{196}D^{197}TSAITQAC^{205}PK<br>LTPLC^{126}VTLQC^{131}TD^{132}VTND^{136}ITDDMR<br>D^{156}C^{157}SFD^{160}MTTELR	7+	1169.8301	1169.8430	11	✗
		8+	1023.7272	1023.7248	2	
		9+	910.0917	910.0868	5	
		10+	819.1832	819.1831	0.2	
	EC ^{153}LK<br>VYSLFYC^{178}LDVVQINENQGNR	2+	1432.1913	1432.1901	1	✓
		3+	955.1300	955.1297	0.3	
		4+	716.5993	716.5988	1	
III	C^{228}K<br>FD^{234}GTGPC^{239}PSVSTVQC^{247}THGIKPVVSTQLLLD^{262}GSLAEEEVMIR<br>VSFEPIPIHYC^{218}APAGFAILK	5+	1347.0804	1347.0829	2	✓
		6+	1122.7349	1122.7363	2	
		7+	962.4881	962.4910	3	
		8+	842.3030	842.3056	4	
		9+	748.8257	748.8269	3	
IV	NILVQFNTPVQID^{295}C^{296}TRPND^{301}NTR<br>QAHC^{331}D^{332}VSK	2+	1721.8332	1721.8282	3	✓
		3+	1148.2245	1148.2254	1	
		4+	861.4202	861.4218	2	
		5+	689.3376	689.3402	4	
		6+	574.6159	574.6173	2	
V	FADSSGGDLEVTTHSFNC^{378}GGEFFYC^{385}DTSGLFDSTWISDTSVQGSDSTGSDDSITLPC^{418}R<br>C^{445}VSDITGLILTR	4+	1852.5669	1852.5527	8	✓
		5+	1482.2549	1482.2581	2	
		6+	1235.3803	1235.3782	2	
gp41 SOSIP	C^{501}K<br>LIC^{604}C^{605}TNVPWN^{611}SSWSNR<br>DQQLLGIWGC^{598}SGK	3+	1176.8808	1176.8796	1	✓
		4+	882.9124	882.9123	0.1	
		5+	706.5314	706.5316	0.3	
		6+	588.9440	588.9439	0.2	

#4: I184C-E190C

Disulfide Loop Domain	Disulfide-Linked Peptides	BG505 SOSIP.664 I184C-E190C D7324 Tag (#4) CS	Theoretical *m/z*	Experimental *m/z*	Mass Error (ppm)	MS/MS
I	DAETTLFC^{54}ASDAK<br>HNVWATHAC^{74}VPTDPNPQEIHLED^{88}VTEEFNMWK	3+	1719.4427	1719.4484	3	✓
		4+	1289.8338	1289.8386	4	
		5+	1032.0685	1032.0727	4	
		6+	860.2250	860.2308	7	
		7+	737.4796	737.4830	5	
II	NNMVEQMHTDIISLWDQSLKPC^{119}VK*<br>LINC^{196}D^{197}TSAITQAC^{205}PK<br>LTPLC^{126}VTLQC^{131}TD^{132}VTND^{136}ITDDMR*<br>D^{156}C^{157}SFD^{160}MTTELR*	6+	1375.2971	1375.3069	7	✓
		7+	1178.9700	1178.9779	7	
		8+	1031.7247	1031.7332	8	
		9+	917.2005	917.2074	8	
		10+	825.5812	825.5881	8	
	C^{190}YR<br>LDVVQC^{184}NENQGNR	2+	963.9309	963.9335	3	✓
		3+	642.9564	642.9587	4	
		4+	482.4691	482.4714	5	
III	C^{228}K<br>FN^{234}GTGPC^{239}PSVSTVQC^{247}THGIKPVVSTQLLLN^{262}GSLAEEEVMIR<br>VSFEPIPIHYC^{218}APAGFAILK	5+	1346.6868	1346.6839	2	✓
		6+	1122.4069	1122.4045	2	
		7+	962.2069	962.2063	1	
		8+	842.0570	842.0566	0.4	
		9+	748.6070	748.6049	3	
IV	NILVQFNTPVQID^{295}C^{296}TRPND^{301}NTR<br>QAHC^{331}D^{332}VSK	2+	1721.8332	1721.8434	6	✓
		3+	1148.2245	1148.2264	2	
		4+	861.4202	861.4227	3	
		5+	689.3376	689.3419	6	
		6+	574.6159	574.6184	4	
V	FADSSGGDLEVTTHSFNC^{378}GGEFFYC^{385}DTSGLFDSTWISDTSVQGSDSTGSDDSITLPC^{418}R<br>C^{445}VSDITGLILTR	4+	1852.5669	1852.5695	1	✓
		5+	1482.2549	1482.2608	4	
		6+	1235.3803	1235.3869	5	
gp41 SOSIP	C^{501}K<br>LIC^{604}C^{605}TNVPWN^{611}SSWSNR<br>DQQLLGIWGC^{598}SGK	3+	1176.8808	1176.8817	1	✓
		4+	882.9124	882.9139	2	
		5+	706.5314	706.5334	3	
		6+	588.9440	588.9450	2	

SI Figure 3

B

Disulfide Loop Domain	Disulfide-linked Peptides	#1	#2	#3	#4
II	LINC^{196}D^{197}TSAITQAC^{205}PK	✓	✓	✓	✓
II	LTPLC^{126}VTLQC^{131}TD^{132}VTND^{136}ITDDMR	✓	Trace	Trace	✓
III	C^{228}K<br>VSFEPIPIHYC^{218}APAGFAILK	Trace	Trace	Trace	Trace
III	FD^{234}GTGPC^{239}PSVSTVQC^{247}THGIKPVVSTQLLLD^{262}GSLAEEEVMIR	✓	✓	✓	✓
gp41 SOSIP	LIC^{604}C^{605}TNVPWN^{611}SSWSNR	✓	✓	✓	✓

SI Figure 4

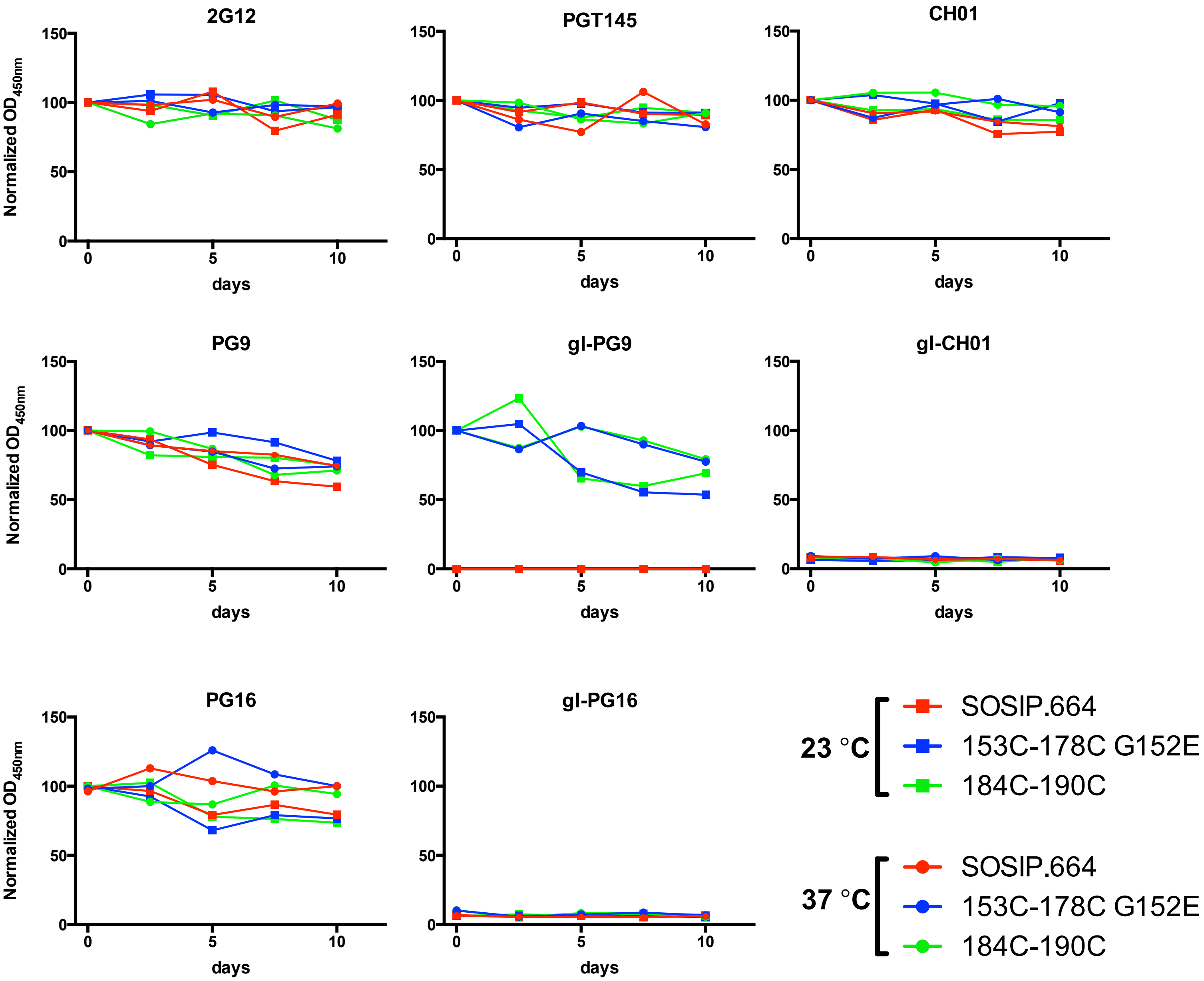

2G12
PGT145
CH01
PG9
gl-PG9
gl-CH01
PG16
gl-PG16
Normalized OD450nm
days
150
100
50
0
0
5
10
23 °C
37 °C
SOSIP.664
153C-178C G152E
184C-190C
SOSIP.664
153C-178C G152E
184C-190C

SI Figure 5

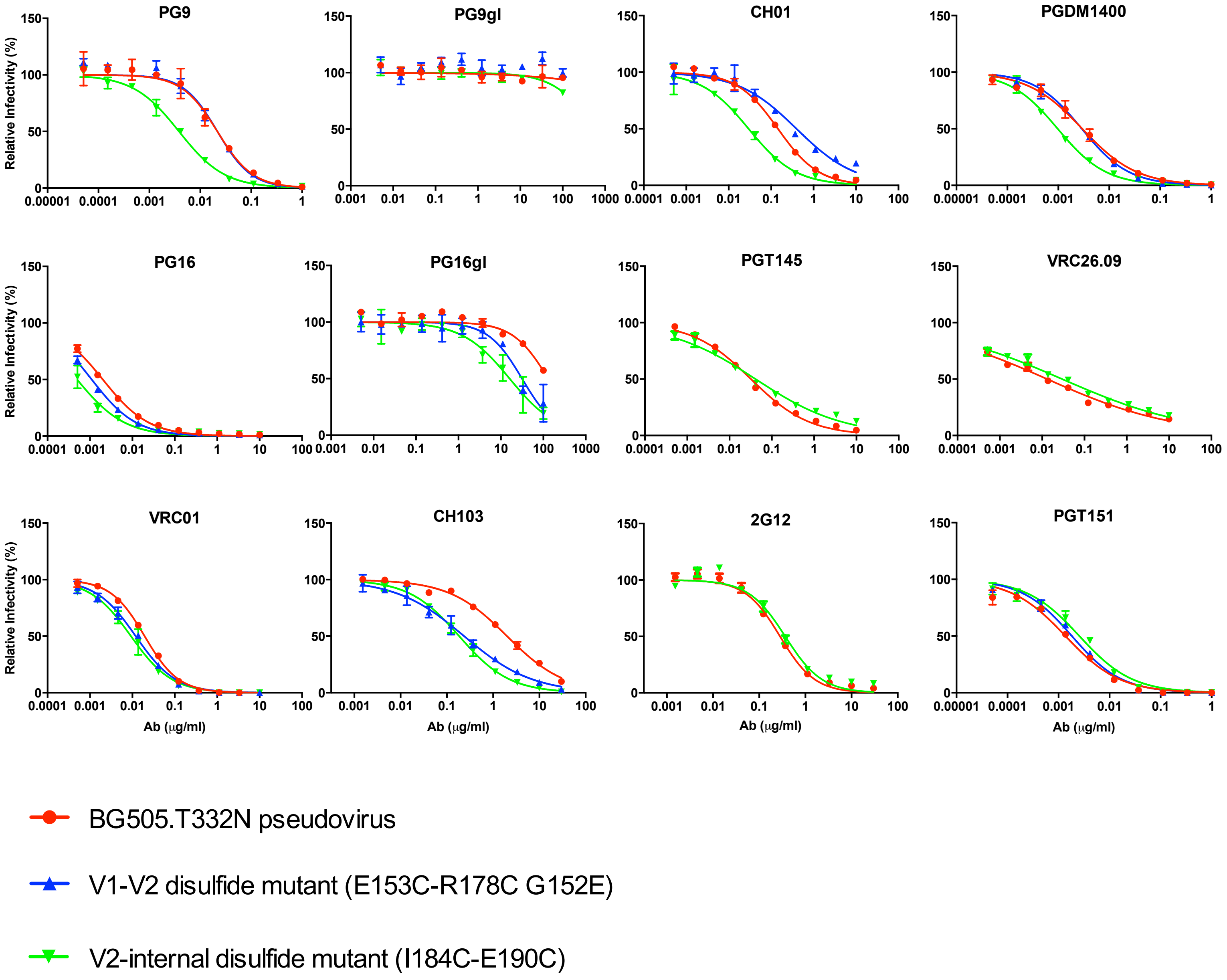
PG9
PG9gl
CH01
PGDM1400
PG16
PG16gl
PGT145
VRC26.09
VRC01
CH103
2G12
PGT151
Relative Infectivity (%)
Ab (μg/ml)
BG505.T332N pseudovirus
V1-V2 disulfide mutant (E153C-R178C G152E)
V2-internal disulfide mutant (I184C-E190C)
